# Polycyclic aromatic hydrocarbons in urban particle matter exacerbate movement disorder after ischemic stroke via potentiation of neuroinflammation

**DOI:** 10.1186/s12989-023-00517-x

**Published:** 2023-02-16

**Authors:** Miki Tanaka, Tomoaki Okuda, Kouichi Itoh, Nami Ishihara, Ami Oguro, Yoshiaki Fujii-Kuriyama, Yu Nabetani, Megumi Yamamoto, Christoph F. A. Vogel, Yasuhiro Ishihara

**Affiliations:** 1 Program of Biomedical Science, Graduate School of Integrated Sciences for Life, Hiroshima University, 1-7-1, Kagamiyama, Higashi-Hiroshima, Hiroshima 739-8521 Japan; 2 Laboratory for Pharmacotherapy and Experimental Neurology, Kagawa School of Pharmaceutical Sciences, Tokushima Bunri University, Sanuki, Kagawa 769-2101 Japan; 3 Faculty of Science and Technology, Keio University, Yokohama, Kanagawa 223-8522 Japan; 4 Medical Research Institute, Molecular Epidemiology, Tokyo Medical and Dental University, Bunkyo, Tokyo 113-8510 Japan; 5 Department of Applied Chemistry, Faculty of Engineering, University of Miyazaki, Miyazaki, 889-2192 Japan; 6 Department of Environment and Public Health, National Institute for Minamata Disease, Minamata, Kumamoto 867-0008 Japan; 7 Department of Environmental Toxicology, University of California, Davis, Davis, CA 95616 USA; 8 Center for Health and the Environment, University of California, Davis, Davis, CA 95616 USA

**Keywords:** Particle matter, Polycyclic aromatic hydrocarbons, Ischemic stroke, Inflammation, Edema

## Abstract

**Background:**

A recent epidemiological study showed that air pollution is closely involved in the prognosis of ischemic stroke. We and others have reported that microglial activation in ischemic stroke plays an important role in neuronal damage. In this study, we investigated the effects of urban aerosol exposure on neuroinflammation and the prognosis of ischemic stroke using a mouse photothrombotic model.

**Results:**

When mice were intranasally exposed to CRM28, urban aerosols collected in Beijing, China, for 7 days, microglial activation was observed in the olfactory bulb and cerebral cortex. Mice exposed to CRM28 showed increased microglial activity and exacerbation of movement disorder after ischemic stroke induction. Administration of core particles stripped of attached chemicals from CRM28 by washing showed less microglial activation and suppression of movement disorder compared with CRM28-treated groups. CRM28 exposure did not affect the prognosis of ischemic stroke in null mice for aryl hydrocarbon receptor, a polycyclic aromatic hydrocarbon (PAH) receptor. Exposure to PM2.5 collected at Yokohama, Japan also exacerbated movement disorder after ischemic stroke.

**Conclusion:**

Particle matter in the air is involved in neuroinflammation and aggravation of the prognosis of ischemic stroke; furthermore, PAHs in the particle matter could be responsible for the prognosis exacerbation.

**Supplementary Information:**

The online version contains supplementary material available at 10.1186/s12989-023-00517-x.

## Background

Air pollution contributed to more than 3% of the annual disability-adjusted life years lost in the 2010 Global Burden of Disease comparative risk assessment, a notable increase since the previous estimate was made in 2000 [1]. A Harvard study of six cities showed that air pollution was positively associated with death from lung cancer and cardiopulmonary disease but not with death from other causes considered together [2], suggesting that the respiratory system is one of the major targets of air pollution. Among several air pollutants, PM2.5 is considered to be one of the major risk factors threatening human health. Inhaled PM2.5 can reach the deep lung and then act on alveolar macrophages and lung epithelial cells to elicit inflammatory reactions and/or oxidative stress. Inflammation is closely associated with oxidative stress: reactive oxygen species inactivate several protein phosphatases, such as PTP1B, which activate a certain kinase pathway to lead to the activation of NF-κB, a master transcription factor for inflammatory gene expression, followed by the upregulation of inflammatory cytokines, chemokines and other molecules [3, 4]. This inflammation and oxidative stress are considered to be involved in asthma aggravation, susceptibility to infection and lung cancer progression [5–7].

Recent evidence has shown that PM2.5 can distribute to organs/tissues other than the respiratory tract. Oberdorster et al. examined the particle distribution in the body by administration of ^13^C carbon nanoparticles to rats and subsequent measurement of the carbon isotope ratio [8]. The ^13^C concentration increased in the lung at the early stage of administration and was also elevated in brain regions such as the cerebral cortex and cerebellum. Interestingly, although ^13^C particles in the lung decreased in a time-dependent manner, those in the brain were not markedly reduced for a long time. Wang et al. revealed that iron oxide nanoparticles reach not only the olfactory bulb but also the cerebral cortex and hippocampus, as shown by the synchrotron radiation microbeam X-ray fluorescence method [9]. Diesel exhaust particles were also reported to move into the olfactory bulb after chamber exposure, as shown by the detection of metals with X-ray fluorescence analysis [10]. In addition, submicrometer-sized polystyrene particles were suggested to pass through the axon and reach the opposite site of neurons [11]. In humans, magnetite nanoparticles were detected within cells in the frontal cortex [12]. Therefore, particle matter introduced by inhalation reaches and accumulates in the brain, although its mechanism is under debate.

Microglia are derived from yolk-sac macrophages, which settle in the brain during development and are the primary immune cells of the central nervous system (CNS). Microglia are activated by several endogenous and exogenous substrates to engulf them and induce inflammatory reactions [13]. Microglia are reported to engulf particles to induce cyclooxygenase-2 (COX-2) expression and release reactive oxygen species [14]. Bacteria and dead cells engulfed by phagocytes are digested to amino acids and other constituents, while silica and carbon, which frequently occupy a PM2.5 core, cannot be digested, and thus many engulfed cells die with the release of alarmins that induce inflammatory reactions [15].

Increasing evidence shows that air pollution is closely involved in neurological disorders. Daily exposure to elevated carbon monoxide concentrations could be associated with an increased risk of epileptic seizures, especially subclinical seizures [16]. Long-term exposure to ambient PM2.5 has increased the risks of death and hospital admissions for Alzheimer's disease [17]. Exposure to black carbon and sulfate in PM2.5 had the strongest associations with dementia incidence [18]. In rats, traffic-related air pollution-exposed animals had more amyloid plaque deposition, higher hyperphosphorylated tau levels, more neuronal cell loss, and greater cognitive deficits [19]. In addition, ambient PM2.5 represents a potentially ubiquitous and modifiable risk factor for the loss of sense of smell [20].

There are several epidemiologic reports showing the relationships between air pollution and the onset of ischemic stroke. It has been suggested that the incidence and hospitalization rates of ischemic stroke are increased in air-polluted areas [21]. Hypertension and atherosclerosis are known risk factors for the onset of cerebral infarction, and PM2.5 is reported to be involved in increases in blood pressure and aggravation of atherosclerosis [22, 23]. Such risk factors affected by PM2.5 may contribute to the increased incidence of stroke due to air pollution. Recently, the relationships between components of PM2.5 and ischemic stroke have been investigated. Zhang et al. reported that the association of ions were generally consistent for ischemic stroke and that polycyclic aromatic hydrocarbons (PAHs), especially indeno(1,2,3-cd)pyrene and benzo(b)fluoranthene, were predominantely associated with ischemic stroke [24]. PAHs upregulate CYP1A1 by an aryl hydrocarbon receptor (AhR)-dependent manner. CYP1A1 is a phase I metabolizing enzyme which plays a key role in metabolic activation of PAHs. Interestingly, CYP1A1 CC genotype, elevated enzyme activity by a T → C mutation, is reported to be associated with 5 times increased risk of ischemic stroke [25]. Therefore, PAHs included in PM2.5 might be one of important determinants for the risk of ischemic stroke.

It has recently become epidemiologically clear that exposure to PM2.5 exacerbates the prognosis of cerebral infarction. PM2.5 exposure has been reported to increase poststroke hospital stay by 2–5 days or increase mortality within 1 year after ischemic stroke [26, 27]. In addition, it was found that the severity of cerebral infarction (NIHSS) was reduced in areas surrounded by green and became more severe in areas with heavy traffic [28]. However, the mechanism by which PM2.5 exacerbates the prognosis of cerebral infarction remains unclear. As mentioned above, PM2.5 can act on the central nervous system and induce inflammatory reactions. Therefore, in this study, we investigated the mechanism by which PM2.5 aggravates the prognosis of cerebral infarction, focusing on microglial activity.

## Results

### Neuroinflammation induced by intranasal exposure to CRM28

CRM28 was collected from filters in a central ventilating system in a building in Beijing and contains several kinds of soluble ions, metals, PAHs and endotoxins (Additional file 1: Tables S3, S4, S5 and S6). The particle size distribution of CRM28 is shown in Additional file 1: Fig. S6. CRM28 included many particles with diameters less than 1 µm.

**Fig. 1:**
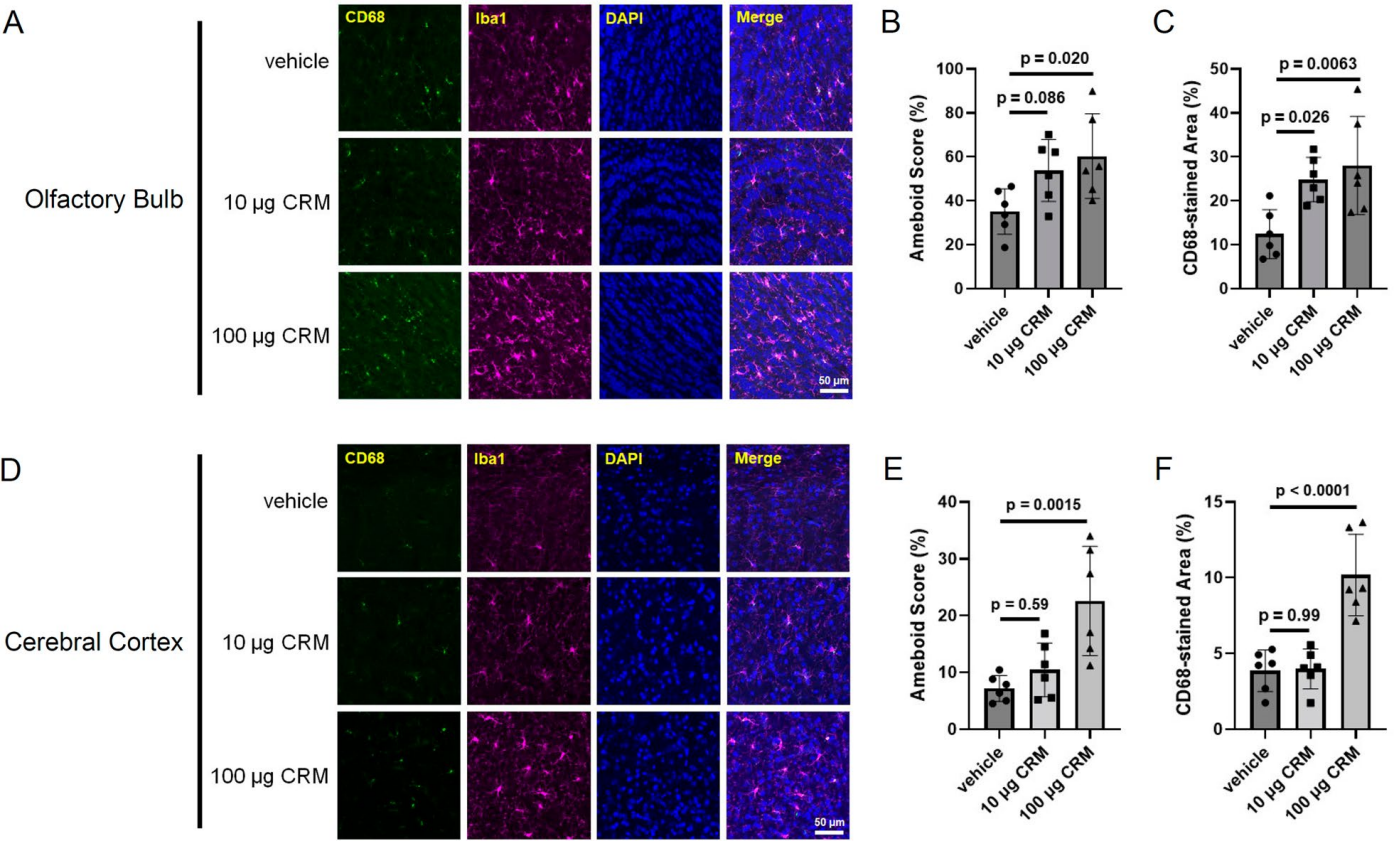
Microglial activation induced by intranasal administration of CRM28. CRM28 was suspended in water and intranasally (i.n.) administered at doses of 0, 10 or 100 µg/mouse once a day for 7 days. Brain slices were stained with Iba1 and CD68 and observed by confocal microscopy. **A** Representative stained pictures of the olfactory bulb. **B** Amoeboid scores. **C** The CD68-stained area of the olfactory bulb. The values are presented as the mean ± S.D. (n = 6 animals in each group). The data were analyzed using one-way ANOVA [**B** F(2, 15) = 4.524, *p* = 0.0290, **C** F(2, 15) = 6.770, *p* = 0.0080] followed by Dunnett’s multiple comparisons test. **D** Representative stained pictures of the cerebral cortex. **E** Amoeboid scores. **F** The CD68-stained area of the cerebral cortex. The values are presented as the mean ± S.D. (n = 6 animals in each group). The data were analyzed using one-way ANOVA [**E**: F(2, 15) = 9.860, *p* = 0.0018, **F**: F(2, 15) = 21.83, *p* < 0.0001] followed by Dunnett’s multiple comparisons test

First, we measured the microglial activity of mice exposed to CRM28 by IHC of Iba1 and CD68 in the olfactory bulb and cerebral cortex because the olfactory bulb is considered to be first target of PM nasally administrated [8] and because ischemia is induced in the cerebral cortex. CRM28 was suspended in the saline and then intranasally administered into mice. Treatment with saline (vehicle) did not affect the expressions of Iba1 and CD68 compared with untreated mice (data not shown). Exposure to 10 µg CRM28 once a day for 7 days did not show microglial morphology evaluated by Iba1 staining or CD68 expression levels in the olfactory bulb and cerebral cortex (Fig. 1). When CRM28 was administered at a dose of 100 µg/mouse once a day for 7 days, an enlarged cell body of microglia and increased expression of CD68 were observed in the olfactory bulb and cerebral cortex (Fig. 1), indicating microglial activation by intranasal exposure to CRM28. The expression of glial fibrillary acidic protein in the cerebral cortex was not altered by the nasal administration of 100 µg CRM28 (Additional file 1: Fig. S1), suggesting that astrocytes could not respond to CRM28. When primary neurons, astrocytes or microglia prepared from mice were treated with 10 µg/mL CRM28, an inflammatory reaction, which is characterized by an increase in inflammatory molecules such as IL-6, KC and COX-2, was strongly induced in microglia, while neurons showed no response, and astrocytes showed less response than microglia (Additional file 1: Fig. S2). In addition, serum cytokine levels were not changed by CRM28 exposure (Additional file 1: Fig. S3), indicating that systemic inflammation did not occur after CRM28 exposure. Therefore, the major target of CRM28 in the brain is considered to be microglia.

### Exacerbation of the ischemic stroke prognosis by CRM28

**Fig. 2:**
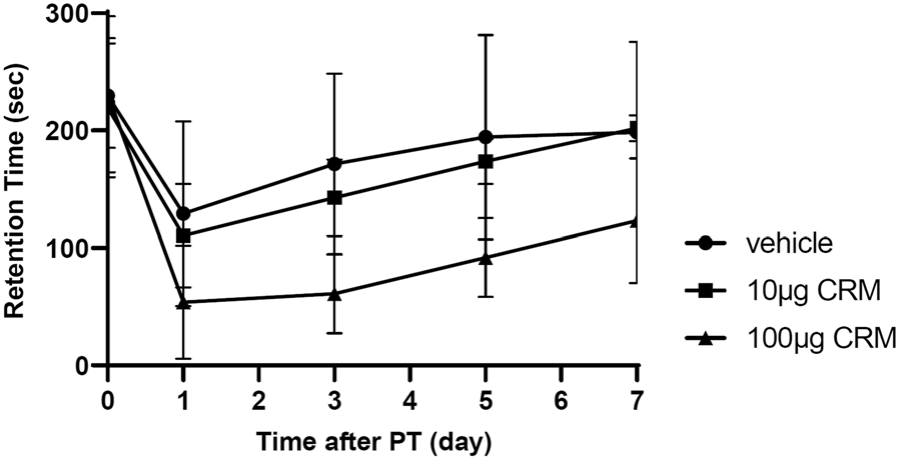
Impairment of movement disorder after ischemic stroke elicited by CRM28 exposure. CRM28 was suspended in water and intranasally (i.n.) administered at doses of 0, 10 or 100 µg/mouse once a day for 7 days. Coordinated movement was measured by a rotarod test before (day 0) and 1, 3, 5 and 7 days after photothrombosis induction. The reported values are the mean ± S.D. (n = 8 animals in each group). The area under the curve (AUC) from 1 to 7 days was calculated, and the AUC was analyzed using one-way ANOVA [F(2, 21) = 8.363, *p* = 0.0021] followed by Dunnett’s multiple comparisons test. The *p* values of the vehicle versus 10 µg CRM groups and vehicle versus 100 µg CRM groups were 0.666 and 0.0018, respectively

Ischemic stroke was induced by cortical photothrombosis with Rose Bengal injection. CRM28 exposure at dosages of 10 and 100 µg/mouse/day for 7 days did not affect mortality by 1 week after ischemic stroke induction (data not shown). Coordinated movement after ischemic stroke was evaluated by rotarod tests. Retention time on the rod 1 day after ischemic stroke induction largely decreased compared with the preischemic period and then gently recovered in a time-dependent manner in the vehicle group (Fig. 2). Preexposure to 10 µg CRM28 for 7 days showed no effect on coordinated movement after ischemic stroke. Administration of 100 µg CRM enhanced the decreases in retention 1 day after induction of ischemic stroke, and notably, the recovery after stroke was delayed by 100 µg CRM 28 exposure (Fig. 2). Treatment with saline did not show the changes in rotarod retention time compared with untreated group (data not shown). These results indicate that CRM28 exposure can exacerbate the prognosis of ischemic stroke.

Edema appears to be critical in the pathogenesis of ischemic stroke, and severe edema can induce exencephaly, leading to a significant aftereffect or occasionally lethality. Recently, we reported that regulation of the progression of ischemic edema by activated microglia is critical for infarct progression and neuronal dysfunction [29]. Therefore, we next examined neuroinflammation, vasogenic edema and infarcts that occur due to ischemia.

**Fig. 3:**
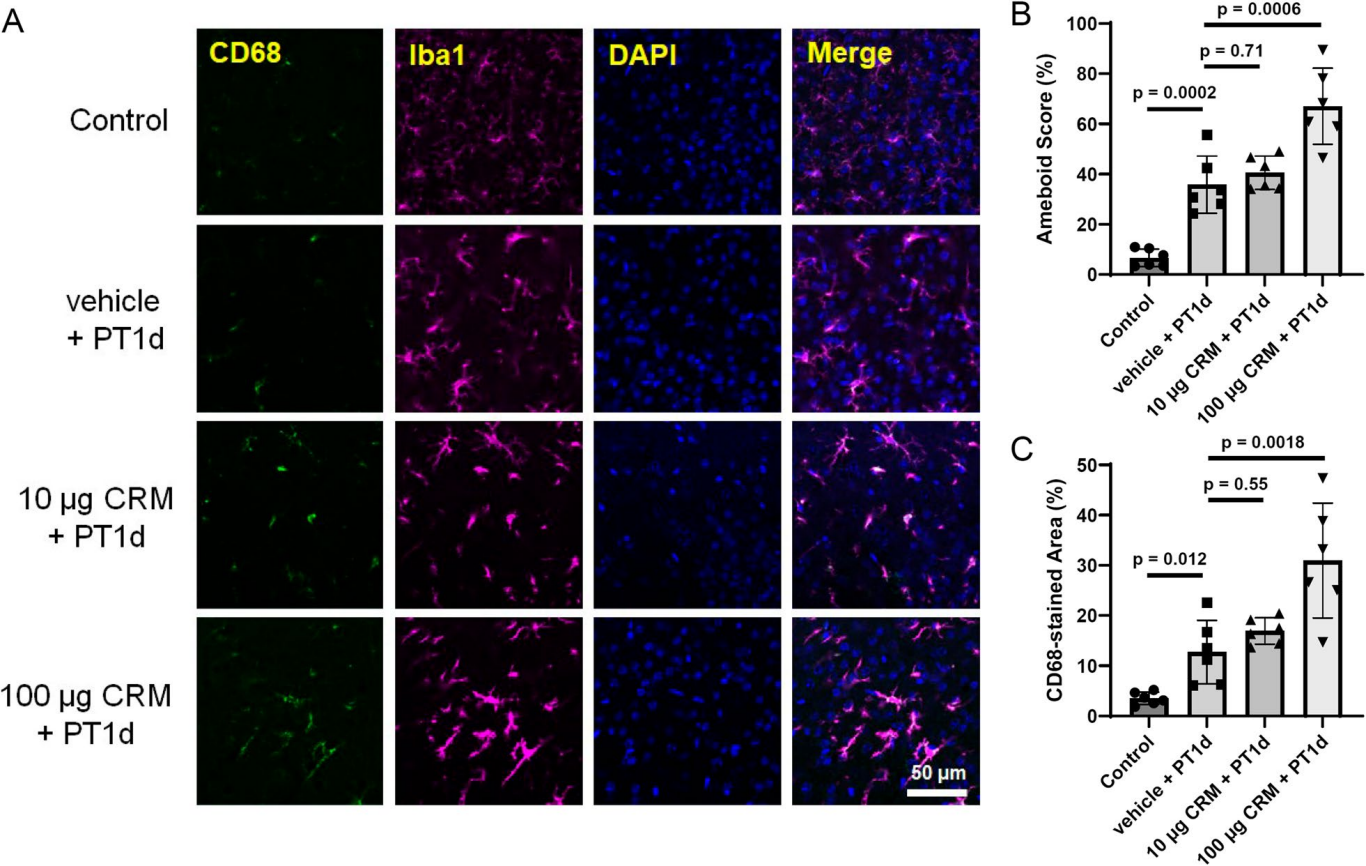
Enhancement of microglial activation during ischemia induced by CRM28 exposure. CRM28 was suspended in water and intranasally (i.n.) administered at doses of 0, 10 or 100 µg/mouse once a day for 7 days. One day after induction of photothrombosis, brain slices were prepared and stained with Iba1 and CD68. The peripheral area of the ischemic area in the cerebral cortex was observed with a confocal microscope. **A** Representative stained pictures of the cerebral cortex. **B** Amoeboid scores. **C** The CD68-stained area of the cerebral cortex. The values are presented as the mean ± S.D. (n = 6 animals in each group). The data between vehicle and vehicle + PT1d were analyzed using Student’s t test. The data among vehicle + PT1d, 10 µg + PT1d and 100 µg + PT1d were analyzed using one-way ANOVA [**B**: F(2, 15) = 12.57, *p* = 0.0006, **C**: F(2, 15) = 9.223, *p* = 0.0024] followed by Dunnett’s multiple comparisons test

**Fig. 4:**
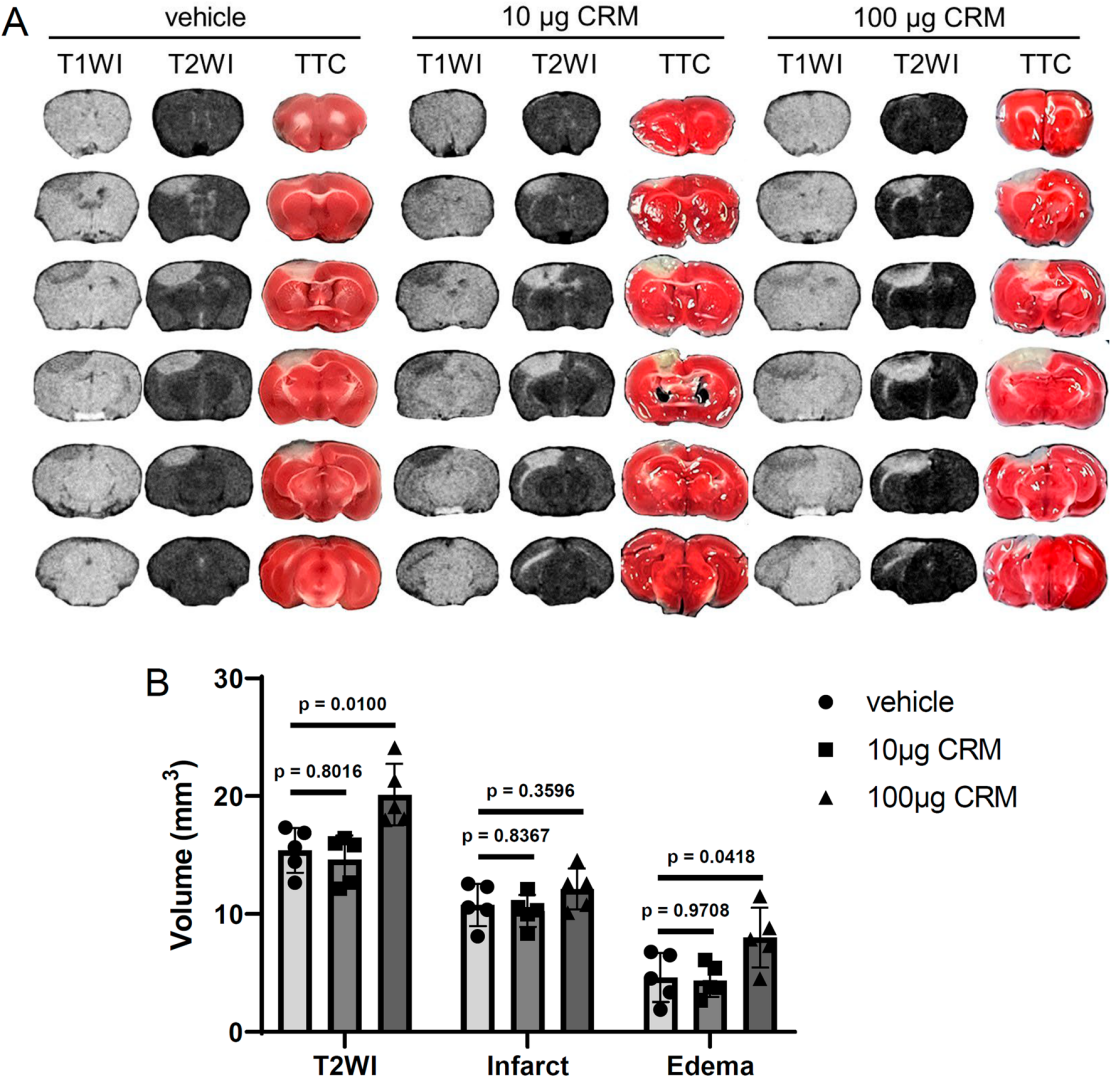
Enhancement of brain edema after ischemic stroke by CRM28 exposure. CRM28 was suspended in water and intranasally (i.n.) administered at doses of 0, 10 or 100 µg/mouse once a day for 7 days. One day after induction of photothrombosis, T1WI and T2WI were obtained for each mouse by MRI, and then the infarct area was determined by TTC staining. **A** Representative images of T1WI, T2WI and TTC staining. **B** T2 hyperintense lesion (T2WI), TTC-unstained area (Infarct) and edema region (T2 hyperintense lesion except infarct) were quantified by ImageJ software. The values are presented as the mean ± S.D. (n = 5 animals in each group). The data were analyzed using one-way ANOVA [T2WI F(2, 12) = 9.124, *p* = 0.0039, Infarct F(2, 12) = 1.708, *p* = 0.2225, Edema F(2, 12) = 4.893, *p* = 0.0279] followed by Dunnett’s multiple comparisons test

The cell body of microglia in the peripheral area of the ischemic region enlarged a day after ischemic stroke induction, as evaluated by Iba1 staining (Fig. 3A, B). CD68 expression also increased 1 day after ischemic stroke (Fig. 3A, C), clearly indicating ischemic activation of microglia. Although exposure to 10 µg CRM28 did not show any change in microglial morphology or CD68 expression, 100 µg CRM28 significantly potentiated the morphological change in microglia from ramified to ameboid and the increase in CD68 expression elicited by ischemia (Fig. 3). Therefore, additive effects of neuroinflammation by CRM28 exposure and ischemia are considered to be observed in the peripheral area of the ischemic region. Magnetic resonance imaging (MRI) T2WIs can be used to visualize both the infarct and edema regions, and 2,3,5-triphenyltetrazolium chloride (TTC) staining can identify the infarct. Thus, the size of the edema region can be determined by calculating the difference in the size of the T2WI hyperintense region from that of the TTC-unstained area [30]. A high contrast area by T2WI appeared, and an infarct also occurred a day after ischemic stroke induction, and the difference between both was considered to be the edema area (Fig. 4). Exposure to 10 µg CRM did not affect the areas of T2-high contrast and TTC staining after ischemia, but 100 µg CRM28 significantly increased the T2 high contrast area and ischemic edema (Fig. 4). Importantly, TTC-stained areas did not change among the vehicle, 10 µg and 100 µg CRM28-treated groups (Fig. 4), indicating that CRM28 exposure does not affect infarct size. Blood pressure and blood coagulation showed no change during CRM28 exposure (Additional file 1: Figs. S4 and S5). Thus, hemodynamics is unlikely to be involved in edema formation. Overall, 100 µg CRM28 exposure for 7 days can enhance ischemic neuroinflammation, which might induce the potentiation of ischemic edema, followed by exacerbation of movement disorder after ischemic stroke.

### Contribution of PAHs in CRM28 to enhanced neuroinflammation and aggravated prognosis after ischemic stroke

**Fig. 5:**
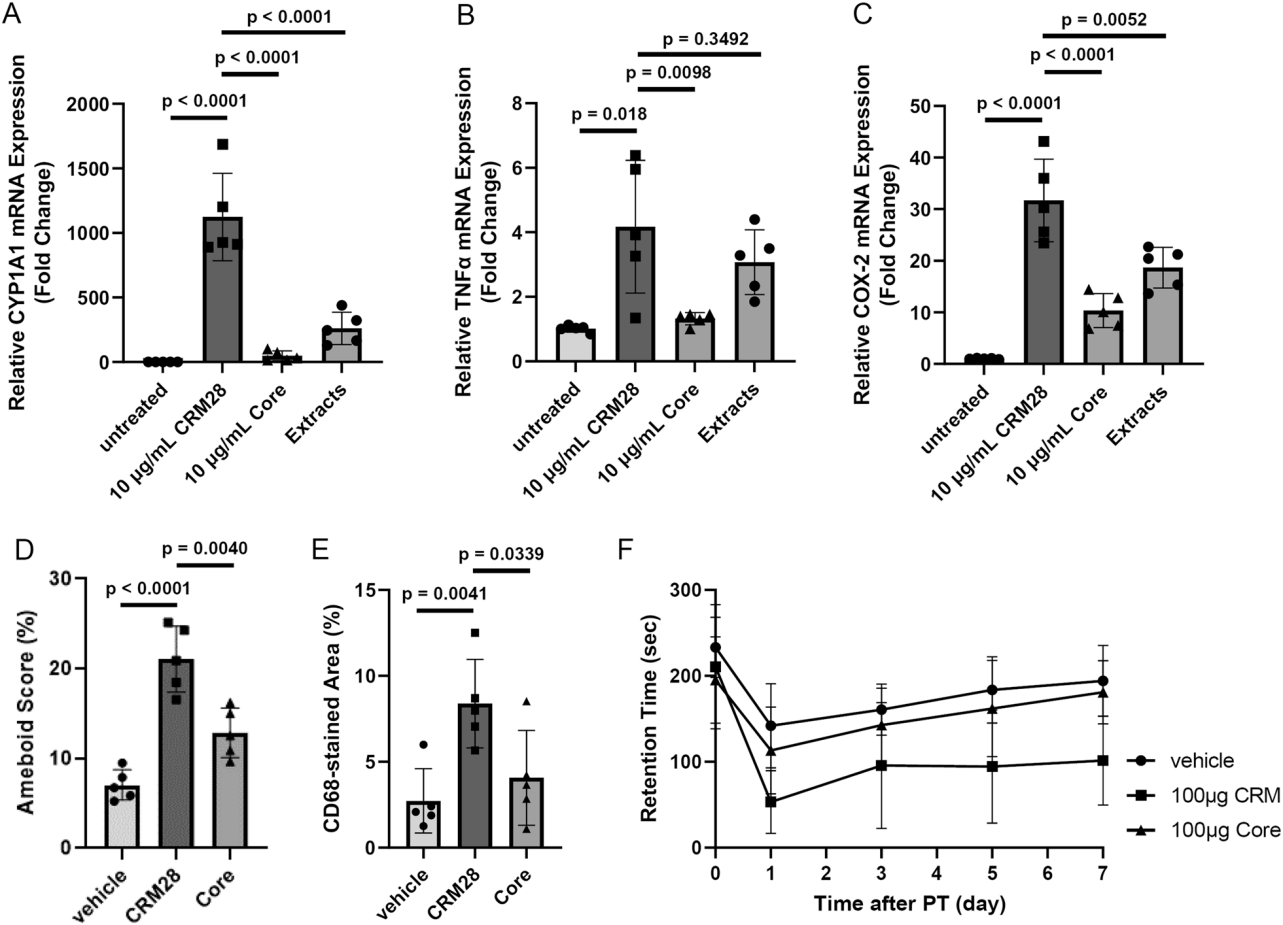
Contribution of attached compounds around CRM28 to neuroinflammation and movement disorder after ischemic stroke. Bone marrow-derived macrophages were treated with 10 µg/mL CRM28 or its core for 6 h, and then mRNA expression of **A** CYP1A1, **B** TNFα and **C** COX-2 was measured by real-time PCR. The values are presented as the mean ± S.D. (n = 5). The data were analyzed using Student’s t test (vehicle group vs. CRM28-treated group) and one-way ANOVA [**A** F(2, 12) = 36.85, *p* < 0.0001, **B** F(2, 12) = 5.875, *p* = 0.0166, **C** F(2, 12) = 19.19, *p* = 0.0002] followed by Dunnett’s multiple comparisons test. Multiple comparisons were corrected by Holm’s method. CRM28 or its core particle was suspended in water and was intranasally (i.n.) administered at a dose of 100 µg/mouse once a day for 7 days. Brain slices were prepared and stained with Iba1 and CD68, and then **D** amoeboid scores and **E** the CD68-stained area of the cerebral cortex were calculated. The values are presented as the mean ± S.D. (n = 5). The data were analyzed using Student’s t test with Holm’s correction for multiple comparisons. **F** After 7 days of exposure to CRM28 or its core, coordinated movement was measured by a rotarod test before (day 0) and 1, 3, 5 and 7 days after photothrombosis induction. The reported values are the mean ± S.D. (n = 9 animals in each group). The area under the curve (AUC) from 1 to 7 days was calculated, and the AUC was analyzed using Student’s t test with Holm’s correction for multiple comparisons. The *p* values of the vehicle versus 100 µg CRM groups and 100 µg CRM versus 100 µg Core groups were 0.0016 and 0.0342, respectively

We next investigated which component in CRM28 is involved in neuroinflammation and the potentiation of movement disorder after ischemic stroke. CRM28 was washed with water and organic solvents to produce core particles. Particle size distribution was not affected by washing procedure (Additional file 1: Fig. S6). The contents of water-soluble ions in the core were much less than those in CRM28 (Additional file 1: Table S3), although the metal concentrations in the core were very similar to those in CRM28 (Additional file 1: Table S4). Of note, the PAH contents in the core decreased to approximately one-tenth of the concentration in CRM28 after washing (Additional file 1: Table S5). Endotoxin contents were also reduced by washing (Additional file 1: Table S6). Bone marrow-derived macrophages were prepared from ICR mice and then exposed to CMR28, core particles or dichloromethane extracts to examine the potential for inducing inflammation. CYP1A1 expression largely induced by CRM28 was significantly suppressed by treatment with core (Fig. 5A), reflecting the decreases in PAH contents in core particles. This consideration is supported by the results that the extracts induced CYP1A1 expression (Fig. 5A). Although TNFα and COX-2 expression was largely induced by treatment with CRM28, their expression was significantly suppressed by core administration (Fig. 5B, C). The extracts had higher potency to increase TNFα and COX-2 expression than core particles (Fig. 5B, C). Therefore, the core lost the potency to induce inflammation by washing compared with CRM28. When mice were intranasally administered 100 µg core particles for 7 days, microglial morphology did not change, and CD68 expression remained low (Fig. 5D, E), indicating that microglia were not affected by core particles. Therefore, chemical compounds were attached to CRM28 and compounds detached from CRM28 by the washing procedure can induce microglial activation by intranasal administration as CRM28. Then, the mice were treated with core particles for 7 days and then subjected to ischemic stroke. Interestingly, the severity of movement disorder in mice treated with the core after ischemic stroke was similar to that in vehicle-treated mice. Therefore, compounds attached to CRM28 could be involved in the aggravation of prognosis after ischemic stroke.

**Fig. 6:**
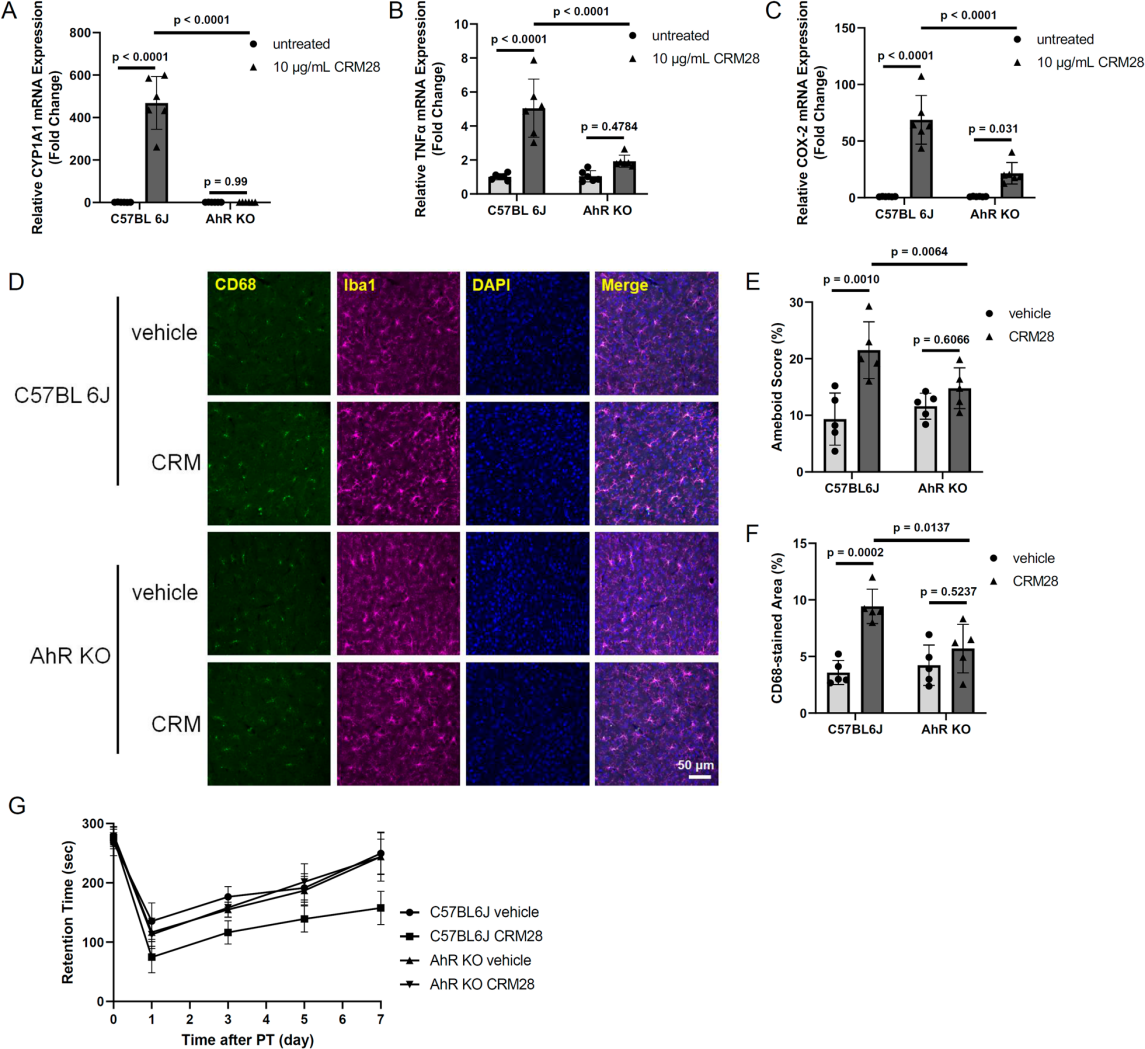
Significant role of PAHs included in CRM28 in neuroinflammation and movement disorder after ischemic stroke. Bone marrow-derived macrophages prepared from wild-type and AhR KO mice were treated with 10 µg/mL CRM28 for 6 h, and then mRNA expression of **A** CYP1A1, **B** TNFα and **C** COX-2 was measured by real-time PCR. The values are presented as the mean ± S.D. (n = 6). The data were analyzed using two-way ANOVA [CYP1A1, F(1, 20) = 84.99, *p* < 0.0001; TNFα, F(1, 20) = 18.55, *p* = 0.0003; COX-2, F(1, 20) = 24.23, *p* < 0.0001] with Tukey's corrected multiple comparison tests. CRM28 was suspended in water and intranasally (i.n.) administered at a dose of 100 µg/mouse once a day for 7 days, followed by staining of the cortex region with Iba1 and CD68. **D** Representative stained pictures of the cerebral cortex. **E** Amoeboid scores. **F** The CD68-stained area of the cerebral cortex. The values are presented as the mean ± S.D. (n = 5). The data were analyzed using two-way ANOVA [**E**: F(1, 16) = 6.251, *p* = 0.0237; **F**: F(1, 16) = 8.507, *p* = 0.0101] with Tukey's corrected multiple comparison tests. **G**. Coordinated movement was measured by a rotarod test before (day 0) and 1, 3, 5 and 7 days after photothrombosis induction. The reported values are the mean ± S.D. (n = 6 animals in each group). The area under the curve (AUC) from 1 to 7 days was calculated, and the AUC was analyzed using two-way ANOVA [F(1, 20) = 25.97, *p* < 0.0001] with Tukey's corrected multiple comparison tests. The *p* values of the wild-vehicle versus wild-CRM groups, AhR KO-vehicle versus AhR KO-CRM groups, and wild-CRM versus AhR KO-CRM groups were < 0.0001, 0.9943 and < 0.0001, respectively

We previously reported that AhR, which is a receptor for PAHs to induce the upregulation of inflammatory molecules, in microglia is involved in neuroinflammation and subsequent brain damage after middle cerebral artery occlusion [30]. In addition, large parts of the PAHs in CRM28 were left by the washing procedure (Additional file 1: Table S5). Thus, we examined the effects of CRM28 on neuroinflammation and movement disorder after ischemia, focusing on PAHs in CRM28. Bone marrow macrophages isolated from wild-type or Aryl hydrocarbon receptor knockout (AhR KO) mice were exposed to 10 µg/mL CRM28 for 6 h. The mRNA expression of CYP1A1, TNFα and COX-2 was increased by CRM28 administration in wild-type macrophages, while in AhR KO macrophages, there was no increase in CYP1A1 mRNA or suppression of TNFα and COX-2 expression in the presence of CRM28 (Fig. 6A–C), suggesting that PAHs in CEM28 contribute to the inflammatory reaction. When wild-type mice or AhR KO mice were exposed to 100 µg CRM28 for 7 days, morphological changes and increased CD68 expression were observed in wild-type mice (Fig. 6D–F). However, microglial cell body enlargement and CD68 expression induced by CRM28 were significantly attenuated in AhR KO mice (Fig. 6D–F). Movement disorder after ischemic stroke induction was also alleviated in AhR KO mice (Fig. 6G). Taken together, these results suggest that PAHs in CRM28 are involved in neuroinflammation and the aggravation of prognosis after ischemic stroke.

### Exacerbation of movement disorder after ischemic stroke by exposure to PM2.5

**Fig. 7:**
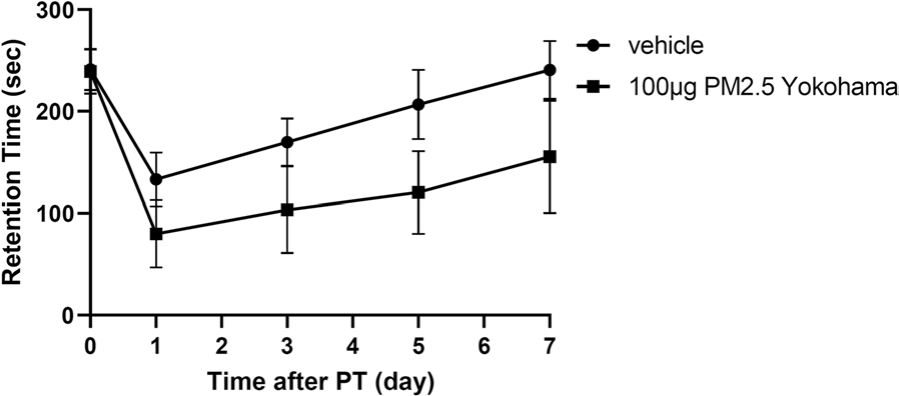
Aggravation of movement disorder after ischemic stroke elicited by PM2.5 collected at Yokohama, Japan. PM2.5 collected at Yokohama, Japan was suspended in water and was intranasally (i.n.) administered at a dose of 100 µg/mouse once a day for 7 days. Coordinated movement was measured by a rotarod test before (day 0) and 1, 3, 5 and 7 days after photothrombosis induction. The reported values are the mean ± S.D. (n = 7 animals in each group). The area under the curve (AUC) from 1 to 7 days was calculated, and the AUC was analyzed using Student’s t test. The *p* value of the vehicle versus 100 µg PM2.5 groups was 0.0013

Finally, we confirmed the generality of the phenomenon: CRM28-induced exacerbation of movement disorder after ischemic stroke. PM2.5 was collected in spring 2020 at Yokohama, Japan, and was introduced to ICR mice at a dose of 100 µg for 7 days. Movement disorder after ischemic stroke induction was alleviated compared with that in vehicle-treated mice (Fig. 7). PM2.5 collected in winter 2018 at Yokohama, Japan showed the exacerbation of movement disorder after ischemia (data not shown). Therefore, it is considered that PM2.5 exposure has the potential to aggravate the prognosis after ischemic stroke.

## Discussion

We have shown in this study that intranasal exposure to CRM28 caused neuroinflammation characterized by microglial activation. Induction of ischemia by photothrombosis after CRM28 exposure exacerbated postischemic neuroinflammation and motor dysfunction compared with the vehicle-treated group. Analysis using both the CRM28 core, in which chemical substances attached to CRM28 were removed, and AhR KO mice indicated that PAHs contained in CRM28 caused enhanced neuroinflammation and exacerbation of motor dysfunction after ischemia. We have reported that ischemia can induce inflammation [29] and that at least part of ischemic inflammation is AhR-dependent [30]. Neuroinflammation has been reported to exacerbate neuropathy due to cerebral infarction [31]. Therefore, CRM28-induced neuroinflammation is considered to act additively or synergistically with ischemia-induced inflammation to exacerbate postischemic motor dysfunction.

The results we present here suggest that any inflammatory substance included in PM2.5 exacerbates the prognosis of ischemic stroke. In addition to PAHs, potent AhR agonists such as polychlorinated dibenzo-p-dioxins (PCDD) and polychlorinated biphenyls (PCB) were detected in the atmosphere, albeit in small quantities in China and Japan [32, 33]. Therefore, these molecules might contribute AhR-dependent neuroinflammation. Ma et al. reported that binding of benzo[a]pyrene at environmentally relevant exposure concentrations altered cytotoxicity to macrophages and they discussed the bioaccessibility of contaminants being carried [34]. In this study, core did not affect neuroinflammation, suggesting that interaction of PAHs with core is important for neuronal damages. Furthermore, it is known that PM2.5 contains endotoxin as a component of bioaerosol. The amount of endotoxin in CRM28 is small compared with that in PM2.5 collected recently [35], probably because it has been approximately 20 years since it was collected. Therefore, although endotoxin is a strong inflammatory molecule, it is possible that PAHs have a strong effect in this study because of the few endotoxins in CRM28. We showed that urban PM2.5 collected in Yokohama also exacerbated the prognosis of cerebral infarction. This PM2.5 contains many endotoxins compared with CRM28, and we previously reported that not only PAHs contained in urban PM2.5 but also endotoxins induce inflammatory reactions [35]. In addition, we suggested the crosstalk of AhR pathway and Toll-like receptor pathway [36]. Therefore, the deterioration of prognosis observed here may be due to endotoxins in addition to PAHs in urban PM2.5.

The target of PAHs is AhR. AhR is known to be highly expressed in the lungs and skin, which are barrier tissues in the body, and it has also been reported to be expressed in several types of cells in the CNS, including microglia [37]. The activation of AhR plays either anti-inflammatory or proinflammatory roles in different environments. Treatment with indole-3-carbinol, a natural ligand of aryl hydrocarbon receptor, induced an increase in IL-1β expression but suppressed LPS-elicited inflammatory reactions in BV-2 microglia [38]. LPS markedly increases inflammation by inducing AhR expression through activation of RelA and binding of RelA/p50 to an NF-κB binding site identified in the human *ahr* gene promoter [39]. In an experimental autoimmune encephalomyelitis model, CX3CR1^+^ microglia-specific AhR deletion upregulated the activation of proinflammatory astrocytes and aggravated inflammation, indicating that microglial AhR suppresses astrocytic inflammation [40]. On the other hand, in ischemic stroke models, AhR exacerbates infarct formation [41], and downregulated IL-1β and IL-6 expression was detected in AhR KO mice [42]. We previously reported that AhR in microglia is involved in neuroinflammation and subsequent vasogenic edema after middle cerebral artery occlusion [30]. The role of AhR in neuroinflammation is still controversial, but AhR might be proinflammatory, at least in ischemia. Further study is needed to uncover the mechanism of action of PAH-specific activation of AhR and its role in the brain. Because astrocytic activity was not changed by CRM28 exposure, aquaporins in astrocytes are not likely to be involved in edema exacerbation.

Increasing evidence has shown that PM2.5 via inhalation from the mouth and nose can target the CNS. In fact, magnetite pollution nanoparticles have been found in humans [12]. Multiple possibilities have been proposed for the pathway by which PM2.5 enters the brain. Oberdorster et al. found that when rats were nasally exposed to carbon (^13^C) particles with a particle diameter (more precisely, median diameter) of 36 nm, carbon microparticles were detected in all brain regions, including the olfactory bulb, cerebrum and cerebellum, a day after exposure [8]. This is considered to be olfactory nerve transport to the CNS. Namely, it is hypothesized that PMs are taken up into olfactory neurons, reach the olfactory bulb via axonal transport, and then spread throughout the CNS via intraneural spaces. In crab nerves, polystyrene particles with a particle size of 0.5 μm have been imaged moving in the axon at a speed of 20–40 μm per minute [11]. TiO_2_ nanomaterials reportedly delivered through intranasal instillation, penetrated and impaired the brain in a particle size-dependent manner [43]. Fe_2_O_3_ nanoparticles also induced neuropathological changes including microglial activation [9]. Recently, it was reported that PM exposure by breathing can be transported from the lungs to the brain via the bloodstream [44]. The data suggest that up to eight times the number of fine particles may reach the brain by traveling, via the bloodstream, from the lungs than pass directly via the nose. CRM28 and urban PM2.5 were administered nasally in this study. The particles are considered to be transported via the olfactory nerve system because inflammation at the olfactory bulb was detected after CRM28 exposure. However, although systemic inflammation did not occur, lung inflammation was detected in this experimental protocol. Therefore, particles inhaled into the lung might move to the CNS. To address the dynamics of particles, we need to observe PM2.5 in our body. However, there are limitations on observation methods for fine particle cores such as carbon or silica, and it is currently extremely difficult to observe small metals by electron microscopy because of artifacts derived from living animals. Another issue is the determination of the pharmacokinetics of PM2.5 or other fine particles.

In this study, we administered CRM28 nasally at 10 and 100 µg/mouse/day and analyzed its neurological effects. As a result, exposure to 10 µg/mouse/day for 7 days had no effect on neuroinflammation or ischemic stroke prognosis, whereas exposure to 100 µg/mouse/day for 7 days induced neuroinflammation and reduced poststroke locomotion, exacerbating functional impairment compared with vehicle-treated mice. Converting the exposure in this study to the exposure per body weight for 7 days, 10 µg/mouse/day is 1.75 mg/kg. An approximate average value of 40 g mouse weight was used for calculation. In Japan, the environmental standard for PM2.5 stipulated by the Ministry of the Environment is "the daily average value of PM2.5 is 35 μg/m^3^ or less". The ventilation volume of a person with a body weight of 60 kg is calculated to be 0.5 L per breath, thus approximately 14.4 m^3^ per day. Then, it became 8.4 µg/kg, as calculated by the Japanese environmental standard. Regarding this calculation, if PM2.5 is inhaled for 208 days at the maximum value within the environmental standards in Japan, the exposure exceeds the no-observed-adverse-effect level estimated in this study. However, considering that PM2.5 is slowly eliminated from the lung or brain [8, 45], long-term exposure over years may be of concern. On the other hand, although air pollution has regional and seasonal characteristics, there are many areas where PM2.5 in the atmosphere is relatively high throughout the year; India’s average PM2.5 concentration level was 75 to over 85 μg/m^3^ in 2019 [46]. Another report showed that the average concentration (± standard deviation) of PM2.5 was 127 ± 77 μg/m^3^ from 2012 to 2021 in Delhi, India, and the highest concentration of PM2.5 per week was approximately 600 μg/m^3^ [47]. In China, it has been reported that PM2.5 concentrations in Beijing, Jinan, and Shanghai are 125.7 μg/m^3^ (18.6–355.5 μg/m^3^), 115.9 μg/m^3^ (44.2–345.4 μg/m^3^), and 85.1 μg/m^3^ (24.3–232.8 μg/m^3^), respectively [48]. Therefore, the effect of PM2.5 on the nervous system may not be negligible in such areas with high PM2.5 concentrations. Recent progress in research on the effects of PM2.5 on the CNS has been remarkable, and it is necessary to consider how to derive the correlation between exposure and neurotoxicity.

Epidemiological studies have revealed that PM2.5 exacerbates many neurological diseases, including Alzheimer's disease, Parkinson's disease, epilepsy, autism, and dysosmia [20, 49–52]. Although the mechanisms of neurological effects of air pollution and/or PM2.5 are largely unknown, reports of animal experiments are being published recently. Patten et al. maintained Alzheimer's disease model rats in a tunnel with heavy traffic for 14 months to investigate the effects on Alzheimer's disease pathology [19]. Inhaled nanosized particles were translocated into the hippocampus, where more amyloid plaque deposition, higher hyperphosphorylated tau levels and more neuronal cell loss were detected compared with control animals. Notably, PM-exposed animals had more microglial activation than control animals, indicating that PM exposure can enhance neuroinflammation and Alzheimer’s disease pathology. To understand more details of the effects of PM on the brain, further mechanistic studies should be performed.

## Conclusion

We investigated the effects of Beijing airborne particulate CRM28 and urban PM2.5 on cerebral infarction prognosis. Nasal exposure to CRM28 induced neuroinflammation characterized by microglial activation and exacerbated motor dysfunction after ischemic stroke. PAHs, including CRM28, can play a fundamental role in enhanced neuroinflammation and exacerbated movement disorder. Therefore, in areas with severe air pollution, controlling the occurrence of PAHs may lead to the prevention of aggravation of cerebral infarction. The significance of this study is that it identified components of PM associated with exacerbated prognosis for ischemic stroke, and this study could contribute to the creation of an environment in which people can live safely and securely by the establishment of novel environmental standards and/or regulation of emission scientifically.

## Methods

### Mice

All animal procedures were performed in accordance with the Fundamental Guidelines for Proper Conduct of Animal Experiments and Related Activities in Academic Research Institutions under the Jurisdiction of the Ministry of Education, Culture, Sports, Science and Technology, Japan. The Animal Care and Use Committee of Hiroshima University approved the experimental protocols (No. C18-16-4 and C20-33). Male C57BL/6J and ICR mice were purchased from CREA Japan Inc. (Tokyo, Japan). AhR-KO mice were generated as described previously [53]. The mice were maintained on a 12:12 h light/dark cycle (light on from 8:00 a.m. to 8:00 p.m.) and had free access to water and food. The mice were allowed to adapt to the facility for 1 week. We used a simple randomization method [54]. Four to five mice were housed in the same cage. No sample calculation was performed, and the sample size was determined by our previous studies [29, 30, 55]. Any animal was not excluded based on the statistics. Two hundred forty mice were used in this study. N values are included in the figure legends as appropriate.

### Particle matter exposure

National Institute for Environmental Studies (NIES) CRM28, urban aerosols collected on filters in a central ventilating system in a building in the Beijing city center from 1996 to 2005 [56], was purchased from the National Institute for Environmental Studies. CRM28 was suspended in saline with sonication (10 W for 10 s) at concentrations of 10 and 100 µg/10 µL. The resulting suspension of 10 µL was immediately applied into the nasal cavity using a micropipette. Vehicle mice were intranasally administered the same amount of saline.

### Immunohistochemistry (IHC)

IHC was performed according to our previous report [57]. The antibodies that were used are listed in Additional file 1: Table S1. Images were processed using the Zen image acquisition software package (Carl Zeiss, Oberkochen, Germany) and ImageJ software. The soma area was evaluated using images of Iba1 staining, and the amoeboid score was calculated by the following formula: (soma area/entire Iba1-stained area) × 100 (%). The Iba1- and CD68-costained areas were analyzed with ImageJ software (National Institutes of Health, Bethesda, MD, USA).

### Total RNA extraction and qPCR

mRNA levels were determined according to the protocol described in our previous report [58]. The primer sequences are presented in Additional file 1: Table S2. mRNA levels were normalized to the level of the housekeeping gene β-actin, and the values of the treated samples were divided by those of the untreated samples to calculate the relative mRNA levels.

### Preparation of the photothrombosis model

Cortical photothrombosis was induced by Rose Bengal injection and subsequent radiation [59, 60]. Anesthesia was initially achieved with isoflurane at 3.5% and then reduced to 2% during the surgical procedure. Mice were placed in a stereotaxic frame, and then the scalp was incised. Rose Bengal (10 mg/mL in sterile saline, 50 mg/kg) was injected intraperitoneally. The LED (170 mW, 561 nm, light diameter 4 mm, M565L3, Thorlabs Inc. Newton, NJ, USA) was illuminated for 20 min, positioned posterior 2 mm and left 2 mm relative to Bregma (motor and somatosensory area). Decreases in blood flow were determined by laser Doppler flowmetry (Unique Medical Co., Ltd., Tokyo, Japan), and mice with a 70 to 85% decrease in blood flow were used for subsequent experiments. The scalp was sutured, and 1 mL of saline was injected subcutaneously. The mice were allowed to recover in a 25 °C chamber for 1 h prior to returning to their home cage. Mice were provided a dish of moist food in their home cage for the duration of their poststroke survival.

### Rotarod test

The rotarod test was performed according to our previous report [55]. A rotarod apparatus (LE8200, Panlab, Barcelona, Spain) was used to test the ability of mice to coordinate movements. The rotarod test was performed by placing a mouse on a rotating drum and measuring the time each animal was able to maintain its balance walking on top of the rod. The speed of the rotarod accelerated from 4 to 40 rpm over a 5-min period. Mice were given 3 trials with a maximum time of 5 min and a 15 min intertrial rest interval. The average latency from 3 trials was calculated.

### MRI

MRI was performed according to our previous paper [29]. Briefly, mice were anesthetized with isoflurane, and body temperature was maintained at a constant 37 ± 0.2 °C. MRI data were acquired using an MRmini-SA system (DS Pharma Biomedical, Osaka, Japan) consisting of a 1.5-Tesla permanent magnet, a compact computer-controlled console, and a solenoid MRI coil with a 30 mm inner diameter. T2-weighted images (T2WIs) were obtained with the following parameters: repetition time (ms)/echo time (ms) = 2500/69 and number of excitations = 4. To measure the signal intensity on T2WIs, the mean signal intensity was determined using INTAGE Realia Professional software (Cybernet Systems Co., Ltd., Tokyo, Japan) and ImageJ software.

### Staining of the infarct region

The infarct size was assessed by TTC staining [29]. Brains were sliced into 1-mm-thick coronal sections 0.0 mm from bregma and stained with 1% TTC solution in PBS at 37 °C for 10 min. TTC-stained brain sections were analyzed by ImageJ software (National Institutes of Health, Bethesda, MD, USA).

### Washing procedure of CRM28

One milliliter of distilled water was added to 50 mg of CRM28. The mixture was vortexed for 5 min and then centrifuged at 30,000 × g for 5 min. This procedure was repeated twice. Then, one milliliter of acetone was added to the resulting pellet. The mixture was vortexed for 5 min and then centrifuged at 30,000 × g for 5 min. This procedure was repeated twice. Finally, one milliliter of dichloromethane was added to the resulting pellet. The mixture was vortexed for 5 min and then centrifuged at 30,000 × g for 5 min. This procedure was repeated twice. The pellet was dried overnight in a desiccator. After the exact weight of the PM core was measured, 100 µg/10 µL suspension was prepared with distilled water. Dichloromethane was evaporated and reconstituted in DMSO to produce dichloromethane extracts, which were added to cultured macrophages.

### Preparation of bone marrow-derived macrophages

Primary bone marrow progenitor cells were isolated and differentiated as described previously [61]. Briefly, the femurs were isolated under sterile conditions, and bone marrow cells were extracted with an RPMI medium-loaded syringe. The cells were passed through a 30 μm cell strainer, and the supernatant was centrifuged for 5 min at 1000 × g to obtain cells. Bone marrow-derived macrophage differentiation was performed in the presence of granulocyte–macrophage colony-stimulating factor (GM-CSF; 20 ng/mL; PeproTech, Cranbury, NJ, USA) for 7 days.

### Collection of PM2.5

The PM2.5 used in this study was collected in Yokohama, Japan, in spring 2020, as previously described [62]. Briefly, PM2.5 with an aerodynamic diameter of 2.5 μm was separated using an impactor prior to entry into the cyclone device. Subsequently, the cyclone imparted a centrifugal force on the gas stream within a conical-shaped chamber and created a vortex inside the cyclone body.

### Statistics

All data are presented as the mean ± standard deviation (S.D.). All data were analyzed using GraphPad Prism 9 (GraphPad Software, San Diego, CA, USA). Student's t test, one-way ANOVA with Dunnett's corrected multiple comparison tests, and two-way ANOVA with Tukey's corrected multiple comparison tests were used to determine significant differences between the means of two or more independent groups. The F value calculated from ANOVA is described in each figure legend, the *p* value is indicated in each figure or legend, and significance was considered when *p* values were less than 0.05.

## Supplementary Information

**Additional file 1:** Includes Fig. S1 to S6, Table S1 to S5 and related methods.

## Data Availability

All data generated or analyzed during this study are included in this published article and its supplementary information file.

## Abbreviations

AhR: Aryl hydrocarbon receptor
CNS: Central nervous system
COX-2: Cyclooxygenase-2
IHC: Immunohistochemistry
MRI: Magnetic resonance imaging
PAH: Polycyclic aromatic hydrocarbon
T2WI: T2-weighted image
TTC: 2,3,5-Triphenyltetrazolium chloride
