# Supplementary material for: Polycyclic aromatic hydrocarbons in urban particle matter exacerbate movement disorder after ischemic stroke via potentiation of neuroinflammation
Source: Part Fibre Toxicol. 2023 Feb 16;20:6. doi: 10.1186/s12989-023-00517-x (PMC9933276; doi:10.1186/s12989-023-00517-x)

**Additional file**

***Tanaka* et al*.***

**Polycyclic aromatic hydrocarbons in urban particle matter exacerbate movement disorder after ischemic stroke via potentiation of neuroinflammation.**

**Methods**

**Analysis of CRM28 and its core**

Water-soluble ions in CRM28 and its core were measured by ion chromatography according to our previous report (Atmosphere 13:319, 2022). Metal contents in PM were measured by IDEA Consultants, Inc. (Tokyo, Japan) using inductively coupled plasma‒mass spectrometry. PAHs in PM were determined using GC‒MS (7890A/5957C, Agilent, Palo Alto, CA, USA) according to our previous report (J Toxicol Sci. 47:201-210, 2022) and the contents in PM were presented as ng/mg PM. Endotoxin levels in the PM were measured using an Endospecy ES-50M set (Seikagaku Co., Tokyo, Japan) according to the manufacturer’s protocol. A control standard endotoxin derived from E. coli O113 (Seikagaku Co., Tokyo, Japan) was used as a standard. The particles were observed by a scanning electron microscope (SU3500 Hitachi High-Tech Science Corporation, Tokyo, Japan).

**Preparation of primary cells**

Mouse primary cortical neurons were prepared from ICR mice on the 17th day of gestation according to our previous paper (J Biol Chem. 290:22805-17, 2015). Briefly, the cortex was dissociated using a papain dissociation system (Worthington Biochemical Corp, Lakewood, NJ, USA). The cells were cultured with neurobasal medium and B27 supplement (Gibco). α-D-Arabinofuranoside was added to inhibit glial proliferation. Cultures of mouse primary astrocytes were prepared from the cerebral cortex of 1–2-day-old male ICR mice (Environ Toxicol Pharmacol. 40:199-205, 2015). The cerebral cortex was treated with 2.5% trypsin and 0.5% DNase I for 20 min at 37 °C. The cells were then plated and cultured with DMEM containing 10% FBS. Mouse primary microglia were prepared from 0- to 1-day-old male ICR mice according to our report (Cells. 10:718, 2021). In brief, the cerebral cortex was dissociated, and the cells were plated with tissue culture medium, which consisted of DMEM supplemented with 10% FBS and 5 µg/mL insulin. After 7 to 10 days of culture, cell dissociation solution including 0.25 U/mL collagenase D (Roche Diagnostics KK, Tokyo, Japan), 8.5 U/mL dispase II (Roche Diagnostics KK), 0.25 U/mL DNase I (Sigma) and 0.1 µg/mL tosyl-L-lysyl-chloromethane hydrochloride) were added to the flask to detach and disperse the cells. Magnetic separation of microglia was performed using the EasySep mouse CD11b Positive Selection Kit II (STEMCELL Technologies, Veritas Corporation, Tokyo, Japan) according to the manufacturer’s instructions.

**Determination of serum cytokine concentrations**

Blood was collected from the tail vein. The serum was isolated, and then cytokine concentrations in the serum were determined with a LEGENDplex Mouse Inflammation Panel (BioLegend) using a CytoFREX S flow cytometer (Beckman Coulter K.K., Tokyo, Japan) according to the manufacturers’ instructions.

**Measurement of blood pressure**

Mice were put into the warmer, which was kept at 39 °C, and the cuff pressure sensor was attached to the tail. Systolic, median and diastolic blood pressures were measured using a BP-98A sphygmomanometer (Bio Research Center Co., Ltd., Nagoya, Japan).

**Assay for blood coagulation**

Blood was collected from the inferior vena cava, and plasma was separated with sodium citrate. Coagulation assays were performed with the blood coagulation analyzer CA-101 (Sysmex Corporation, Kobe, Japan) using kits, Thromborel S (Sysmex Corporation), Actin FSL (Sysmex Corporation) and Multifibern U (Sysmex Corporation) for measurement of prothrombin time, activated partial thromboplastin time and fibrinogen concentration, respectively, according to the manufacturers’ instructions.

**Table S1. Antibodies used in this study**

Target Source RRID Dilution (utility)

Iba1 Wako Pure Chemical AB_839504 1/400 (IHC)

CD68 BIO-RAD AB_322219 1/400 (IHC)

GFAP Agilent (Dako) AB_10013482 1/400 (IHC)

Rat IgG, Alexa 488 TFS AB_2534074 1/200 (IHC)

Rabbit IgG, Alexa 488 TFS AB_10563748 1/200 (IHC)

Rabbit IgG, Alexa 568 TFS AB_10563566 1/200 (IHC)

TFS: Thermo Fisher Scientific, ICH: immunohistochemistry.

**Table S2. Primers used for qPCR**

Name Sequence (5’-3’)

Mouse β-actin-For CTAGGCACCAGGGTGTGATG

Mouse β-actin-Rev GGGGTACTTCAGGGTCAGGA

Mouse CYP1A1-For GGCCACTTTGACCCTTACAA

Mouse CYP1A1-Rev CAGGTAACGGAGGACAGGAA

Mouse TNFα-For ATGGCCTCCCTCTCATCAGT

Mouse TNFα-Rev CTTGGTGGTTTGCTACGACG

Mouse COX-2-For AGCCAGGCAGCAAATCCTT

Mouse COX-2-Rev CAGTCCGGGTACAGTCACAC

Mouse IL-6-For TCCTCTCTGCAAGAGACTTCC

Mouse IL-6-Rev TTGTGAAGTAGGGAAGGCCG

Mouse KC-For CTTGAAGGTGTTGCCCTCAG

Mouse KC-Rev TGGGGACACCTTTTAGCATC

**Table S3. Contents of water-soluble ions in PM**

CRM28 Core

Cl⁻ 5473 n.d.

NO₃⁻ 18040 6961

SO₄²⁻ 81304 46711

Na⁺ 2457 n.d.

NH₄⁺ 2943 1410

K⁺ 2834 n.d.

Mg²⁺ 2878 n.d.

Ca²⁺ 40732 24506 (ppmw)

n.d.: not detected

**Table S4. Contents of metals in PM**

CRM28 Core

Mg 14000 15800

Al 50400 72000

S 39100 14800

K 13700 17100

Ca 66900 54200

Ti 2920 3690

V 73 117

Cr 136 98

Mn 686 667

Fe 29200 40200

Co 22 26

Ni 63 87

Cu 104 141

Zn 1140 692

As 90 110

Se 14 11

Cd 5.6 3.3

Pb 403 517 (ppmw)

**Table S5. Contents of PAHs in PM**

CRM28 Core

Naphthalene 0.004 0.003

Acenaphthylene 0.008 n.d.

Acenaphthene n.d. n.d.

Fluorene 0.015 0.003

Phenanthrene 0.27 0.12

Anthracene 0.026 0.014

Fluoranthene 0.80 0.17

Pyrene 0.50 0.10

Benz(a)anthracene 0.67 0.14

Chrysene 2.8 0.31

Benzo(b)fluoranthene 8.4 0.78

Benzo(k)fluoranthene 3.2 0.31

Benzo(e)pyrene 3.2 0.34

Benzo(a)pyrene 0.98 0.13

Indeno(1,2,3-cd)pyrene 2.2 0.22

Dibenz(a,h)anthracene 0.85 0.064

Benzo(ghi)perylene 2.0 0.22

Coronene 1.0 0.20

Total PAHs 27 3.1 (ng/mg PM)

n.d.: not detected

**Table S6. Contents of endotoxin in PM**

CRM28 Core

Endotoxin 0.090 0.018 (IU/mg PM)

**Figure legends**

**Figure S1. No change in astrocytic activity was observed during CRM28 exposure.**

CRM28 was suspended in water and was intranasally (i.n.) administered at doses of 0, 10 or 100 µg/mouse once a day for 7 days. Brain slices from the cerebral cortex were stained with GFAP and observed by confocal microscopy. Representative images of GPAP and DAPI staining are shown.

**Figure S2. Induction of an inflammatory reaction by CRM28 in primary microglia but not in neurons or astrocytes.**

Mouse primary neurons, astrocytes and microglia were treated with 10 µg/mL CRM28 for 6 h. RNA was isolated, and real-time PCR was performed to detect the expression of (A) IL-6, (B) KC and (C) COX-2. The values are presented as the mean ±S.D. (n = 3). Data were analyzed using Student’s t test.

**Figure S3. No effect of CRM28 exposure on peripheral inflammation.**

CRM28 was suspended in water and was intranasally (i.n.) administered at doses of 0, 10 or 100 µg/mouse once a day for 7 days. The serum was separated, and then the concentrations of IL-2, IL-4, IL6, IL-17A and TNFα were determined with a LEGENDplex Mouse Inflammation Panel. The values are presented as the mean ± S.D. (n = 4).

**Figure S4. There was no change in blood pressure after CRM28 exposure.**

CRM28 was suspended in water and was intranasally (i.n.) administered at doses of 0, 10 or 100 µg/mouse once a day for 7 days. Systolic, median and diastolic blood pressures (SBP, MBP and DBP, respectively) were measured by a noninvasive method. The values are presented as the mean ±S.D. (n = 7).

**Figure S5. No effect of CRM28 exposure on blood coagulation.**

CRM28 was suspended in water and was intranasally (i.n.) administered at doses of 0, 10 or 100 µg/mouse once a day for 7 days. Plasma was separated, and then a series of coagulation assays were performed: (A) prothrombin time, (B) activated partial thromboplastin time and (C) fibrinogen concentration. The values are presented as the mean ±S.D. (n = 8).

**Figure S6. Particle size distribution of CRM28 and its core.**

CRM28 was washed according to the Materials and methods to produce core particles. CRM28 and core particles were observed under a scanning electron microscope. Over 1,000 particles were counted and measured to calculate the relative frequency.

Figure S1


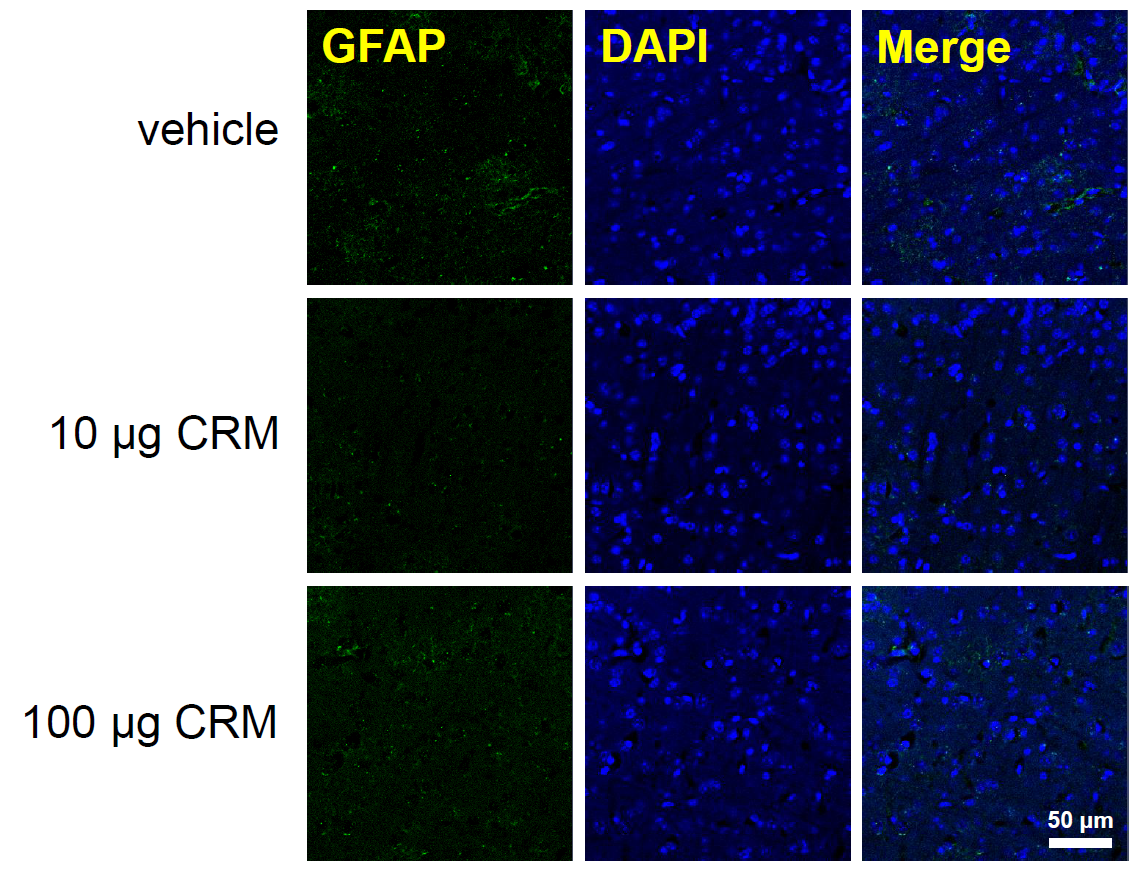


Figure S2


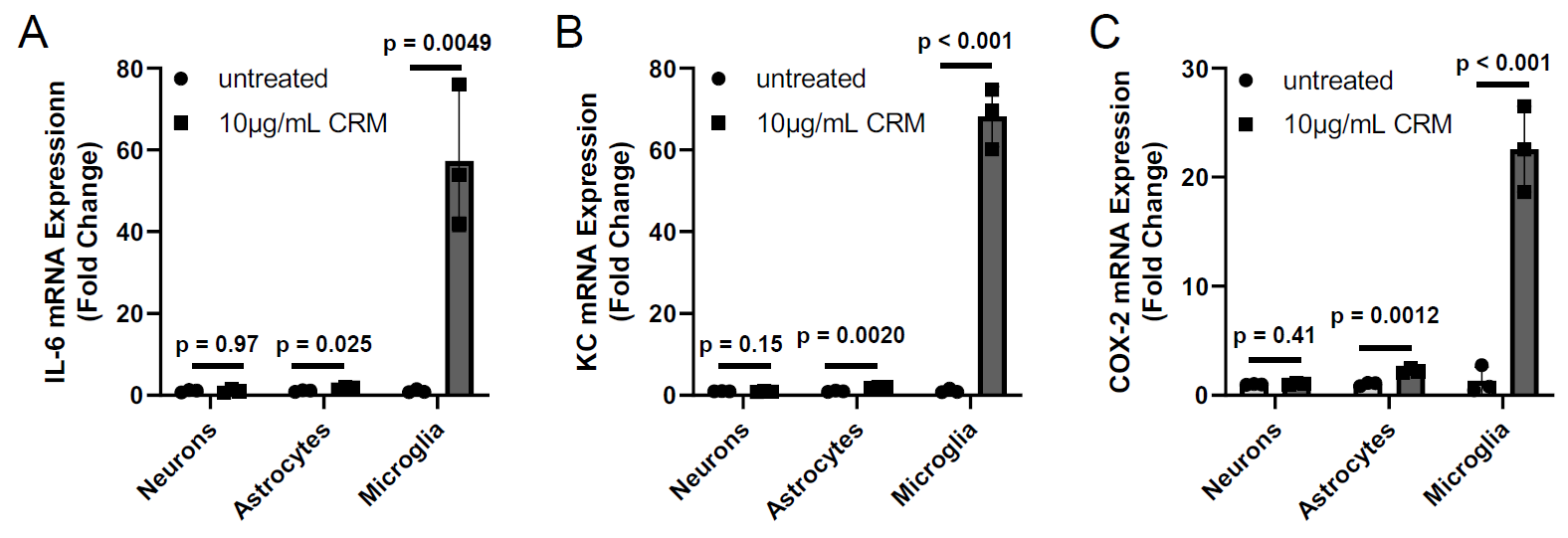


Figure S3


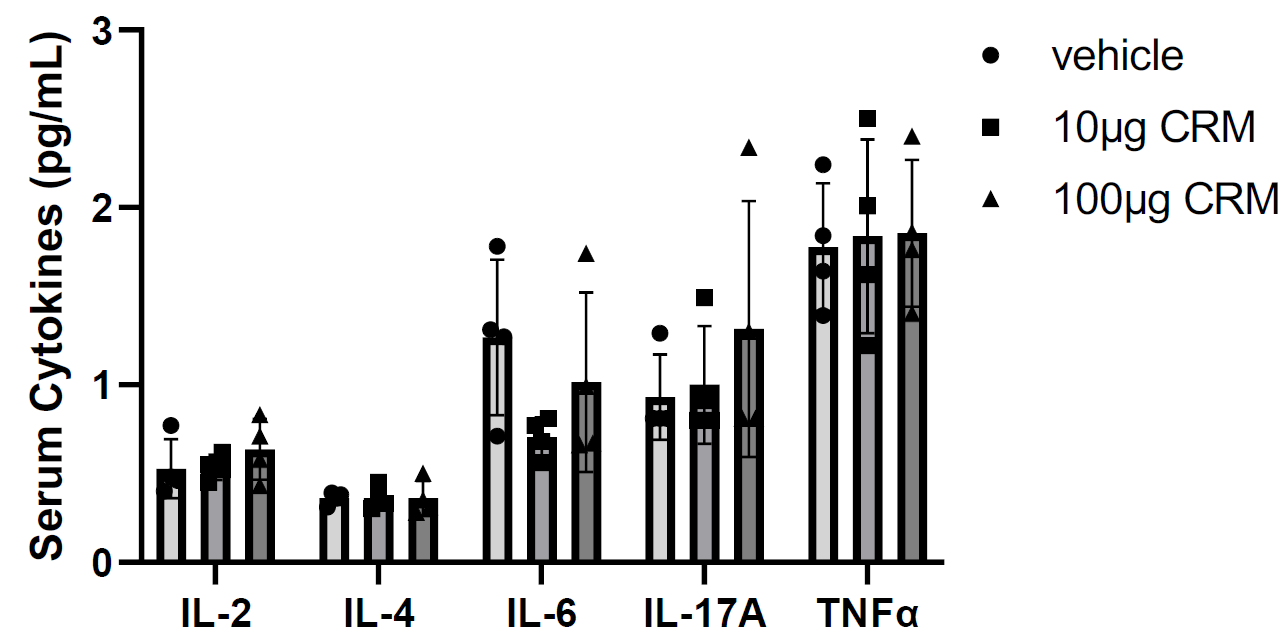


Figure S4


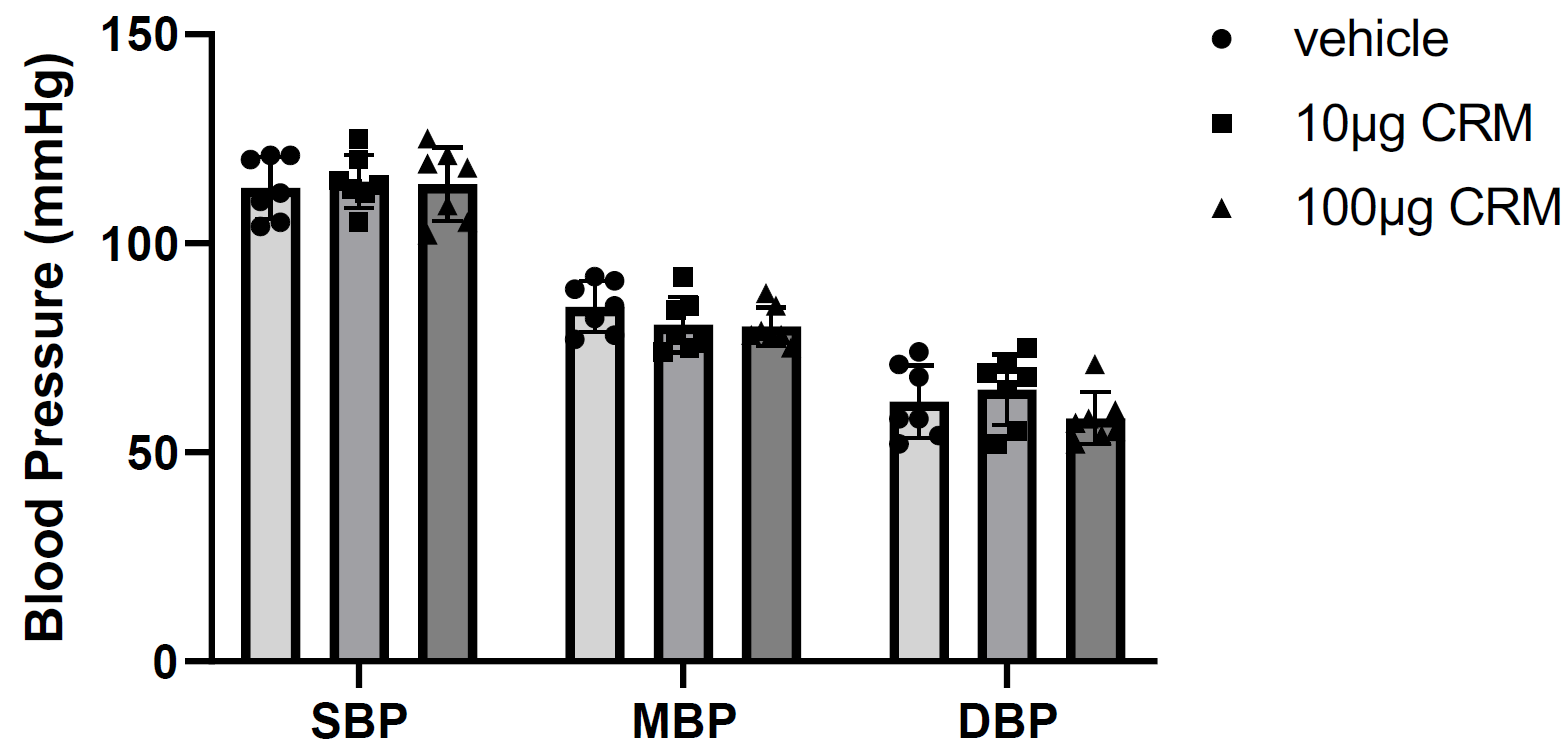


Figure S5


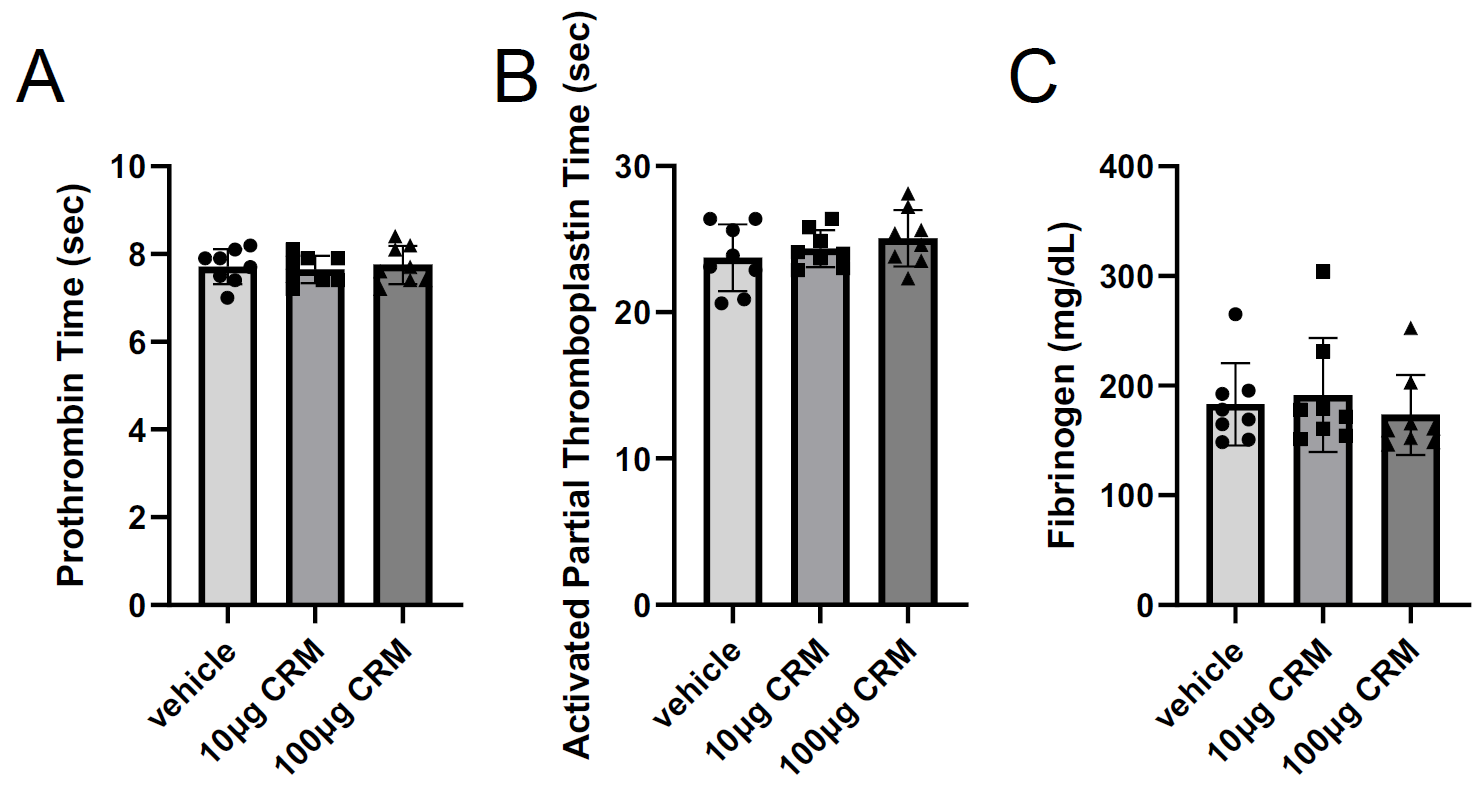


Figure S6


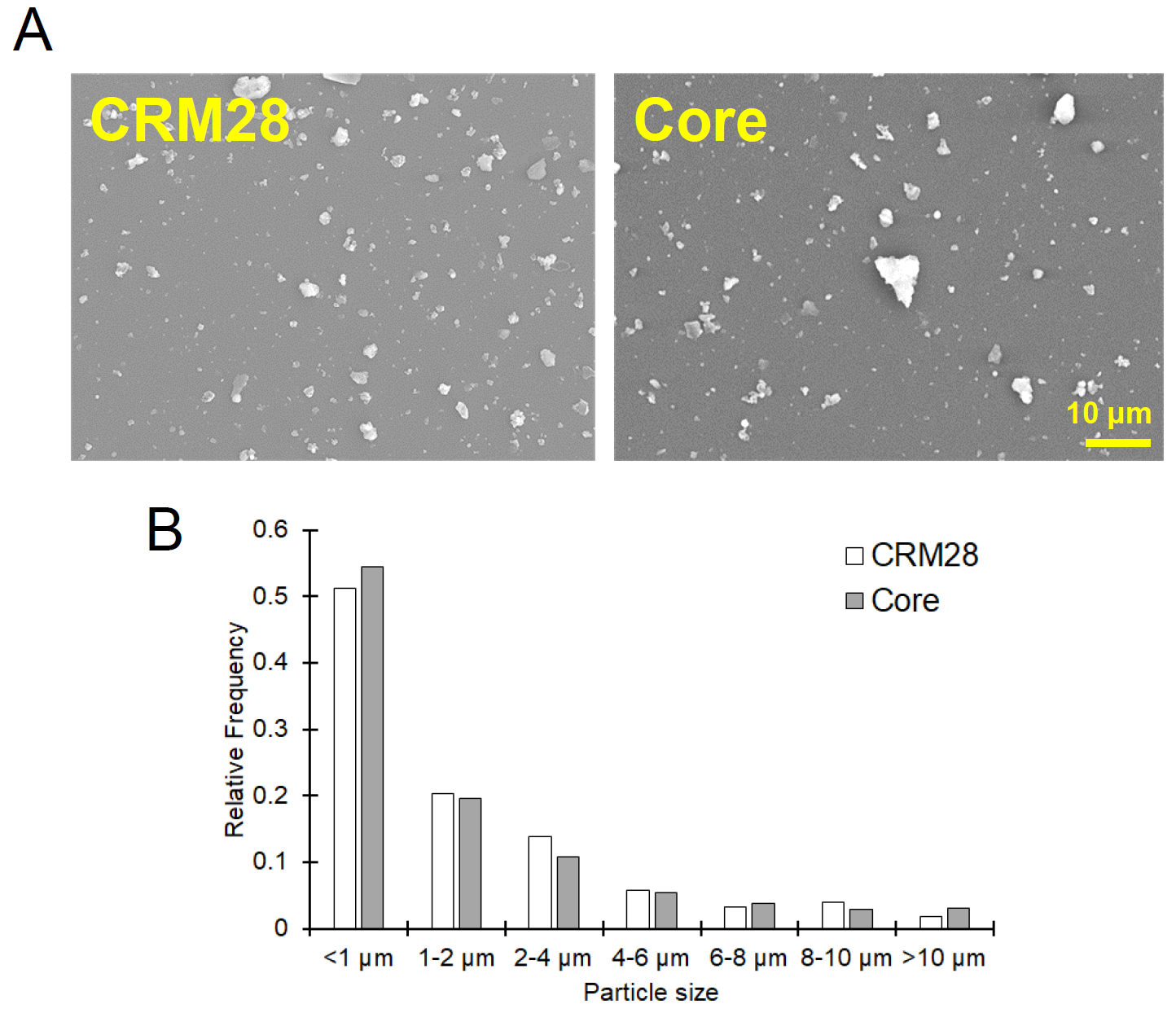

Supplement: Supplementary file 1 — Additional file 1: Includes Fig. S1 to S6, Table S1 to S5 and related methods. [file 12989_2023_517_MOESM1_ESM.docx]
